# Visible-Light-Induced Photocatalytic Degradation of Naproxen Using 5% Cu/TiO_2_, Transformation Products, and Mechanistic Studies

**DOI:** 10.3390/molecules29235752

**Published:** 2024-12-05

**Authors:** Sarah Ahmed Hasan, Abbas Khaleel, Soleiman Hisaindee, Mohammed A. Meetani

**Affiliations:** Chemistry Department, College of Science, United Arab Emirates University, Al-Ain P.O. Box 15551, United Arab Emirates; sarahh10@illinois.edu (S.A.H.); abbask@uaeu.ac.ae (A.K.); soleiman.hisaindee@uaeu.ac.ae (S.H.)

**Keywords:** naproxen, 5%Cu/TiO_2_, visible light, photocatalytic degradation, degradation pathway, degradation mechanism

## Abstract

The presence of drugs in wastewater effluent is of concern due to their effects on the aquatic fauna and flora and there are growing efforts for their removal from the environment. In this paper, we study the photocatalytic visible-light degradation of naproxen, an over-the-counter anti-inflammatory drug, using 5% copper-doped TiO_2_. The photocatalyst was characterized by XRD and BET surface area measurements. The optimal conditions for the degradation of 1 × 10^−3^ M of naproxen were found to be 3 h, with a catalyst loading of 50 mg/100 mL of the drug solution, and an acidic pH of 4.55. The degradation followed pseudo-first order kinetics and achieved a photodegradation efficiency of 44.8%. HPLC was used to separate the degradation products and their structures were determined using MS/MS data. A pathway for the degradation of naproxen is proposed along with degradation mechanisms. The major degradation events involve the formation of hydroxyl radicals, hydroxylation, keto-enol tautomerism, and decarboxylation.

## 1. Introduction

Naproxen is an NSAID (nonsteroidal anti-inflammatory drug) that is widely utilized to relieve and manage pain and inflammation associated with post-surgery procedures and other musculoskeletal conditions. Naproxen is a safer alternative to more potent and addictive drugs such as opioids [1,2]. Naproxen works by blocking cyclo-oxgenase-1 and 2 enzymes in physiological and inflammatory pathways [2,3].

The widespread use of naproxen in the treatment of a multitude of conditions and ailments is not without danger. It can be toxic and detrimental to humans. Naproxen enters the aquatic environment through human excretions, such as urine and feces, and through sewage and wastewater from treatment plants [4,5,6]. For instance, out of every five sewage treatment facilities in China, two were discovered to have naproxen in their effluent and particulate exiting the plants with concentrations ranging from 2.5 to 80.5 ng/L [7]. Studies in Iran showed the presence of naproxen in various water samples from rivers, tap water, and sewage inlets and outlets with concentrations ranging from 0.029 to 214 µg/L [8]. Additionally, in Algeria, samples of effluent from the Beni Messous wastewater treatment plant were shown to have naproxen with a concentration of up to 333.7 ng/L [9].

Naproxen hinders the development and growth of marine species as well as suppressing fish antioxidant gene expression, and it has also been found to be carcinogenic and capable of causing oxidative stress to the human body [4,10]. Naproxen and its degradation products have been shown to have negative ecotoxicological effects on organisms in ecosystems. Bacteria, algae, fish, amphibians, and crustaceans were shown to be undesirably affected by naproxen [11]. For instance, photo-derivatives of naproxen have been found to be toxic to Brachionus calyciflorus, Thamnocephalus platyurus, Ceriodaphnia dubia, Vibrio fischeri, and Daphnia magna [12,13].

In another study, naproxen that had bio-accumulated in the bile of fish was a thousand-fold greater than the concentration in the lake water [14]. It is believed that metabolizing enzyme activity [15] is suppressed. In zebrafish, Ding et al. [16] showed that environmental concentrations of naproxen were sufficient to affect mRNA expression, causing gastrointestinal and renal effects. Moreover, cyclooxygenase (COX) was inhibited, causing tachycardia, pericardial oedema, and teratogenic effects. Thyroid functions were also impaired, decreasing thyroid hormone levels. It is suspected that gene transcription is disrupted in the hypothalamic–pituitary–thyroid axis, resulting in a significant decrease in transthyretin levels [15].

In the flag fish, egg fertilization was decreased after a lengthy exposure to 0.1 μg/L of naproxen. Low concentrations of naproxen also inhibited the growth of crustaceans such as Ceriodaphnia dubia after 7 days of exposure [17].

In algae, naproxen had an EC50 value of 40 mg/L in Chlorella vulgaris and Ankistrodesmus falcatus [16]. In another study by the same authors, the toxicity of naproxen in Cymbella species and Scenedesmus quadricauda was both dose and time-dependent. The inhibition of growth increased with higher concentrations of naproxen but decreased with longer durations of the exposure. Moreover, decreases in chlorophyll a, chlorophyll b, and carotenoids were also observed. Naproxen was also responsible for a reduction in enzymatic antioxidants, which led to an accumulation of OH− and H_2_O_2_. At the same time, there was an increase in the concentration of malondialdehyde, which may interact with proteins, lipoproteins, and DNA.

Garcia-Medina et al. [18] showed enhanced superoxide dismutase activity in Hyalella Azteca after incubation with naproxen, which is related to the generation of reactive oxygen species, leading to nucleic acid damage.

In other studies, the synergistic effects of naproxen with other contaminations were investigated. Melvin et al. [19] demonstrated a negative and interactive effect of naproxen, carbamazepine, and sulfamethoxazole on the growth and development of the amphibian Limnodynastes peronei at environmental concentrations. Jiang et al. [20] showed that a mixture of naproxen, diclofenac, and ibuprofen led to an increase in bacterial diversity, with an enrichment in Actinobacteria and Bacteroidetes and a decrease in Micropruina and Nakamurella. Moreover, they observed damage in the cell wall of the microorganisms.

Grenni et al. [21] showed that the chronic exposure to naproxen seen in the natural microbial community of the Tiber River caused a decrease in β-Proteobacteria (ammonia-oxidizing bacteria) and Archaea. However, an increase in the population of α- and γ-Proteobacteria was observed. The latter are thought to be involved in the biodegradation of naproxen.

Consequently, the removal of naproxen from wastewater, before it reaches water bodies and sources, is a priority [7]. Naproxen is not readily biodegradable but can be degraded under visible-light conditions in the presence of a photocatalyst [22]. There have been many studies regarding the photodegradation of naproxen with a diverse set of inorganic and nano catalysts [13,23,24,25,26,27,28]. Reduced graphene sheet functionalized with bismuth oxide silicates and carbon nitrides (rGO/BSO/g-C_3_N_4_) along with visible-light exposure of an hour and a half, resulted in an removal efficiency of 77.5% of naproxen from wastewater samples [29]. In another study nanosheets (Bi_2_MoO_6_/g-C_3_N_4_ with a 50/50 mass ratio) doped with molybdenum showed increased photodegradation efficiency of 83% [23]. A graphene sheet with carbon nitride as functional groups (g-C_3_N_4_) was used to photodegrade naproxen and its degradation products from LC-MS/MS studies, included 2-methoxy-6-vinylnapthelene, 2-(napthalen-2-yl)propanal, and 1-(6-methoxynapthalen-2-yl)ethan-1-one [27]. TiO_3_^2−^ nanobulks altered with bismuth was effective in removing naproxen at a pH of 7 with about 99.9% efficacy [24]. A nano heterocomposite catalyst of gamma-iron oxyhydroxide (γ-FeOOH) was used in conjunction with a UV photochemical reactor with an optimized pH of 5, displaying first-order kinetics, and by-products of dimethyl benzene-1,2-dicarboxylate, 2-propylbenzoic acid, 1-methoxy-6-methylnapthalene, succinic acid, among others [28].

Other than nano catalysts, there are also inorganic catalysts, like titanium dioxide, that have been described in several studies to have remarkable results in degrading naproxen with UV stimulation [13,25,26]. To date, there are very limited studies on the photodegradation of drugs, especially naproxen and specifically by copper-doped titanium dioxide sheets (CuTiO_2_) [30,31,32,33]. For instance, CuTiO_2_ assisted photodegradation was performed on drugs other than naproxen, such as ketoprofen, meloxicam, and tenoxicam. The CuTiO_2_ photocatalyst was used in combination with other components, such as hydrozincite, alumina, and fluoride [30,31,32,33]. The closest study (CuTiO_2_, calcinated at 600 °C) to the current approach, uses UV radiation and the photodegradation products of naproxen were not identified [32]. It mainly depicted the characterization of differently doped titanium dioxide sheets and compared them with regards to their degradation efficacy for multiple drugs [32].

Therefore, in this study, 5% CuTiO_2_ is used in the presence of visible light for the optimal photodegradation of naproxen along with a study of the degradation products by LC-MS/MS. A degradation pathway is proposed with plausible mechanisms.

## 2. Results and Discussion

### 2.1. Catalyst Characterization and XRD Analysis

**Figure 1:**
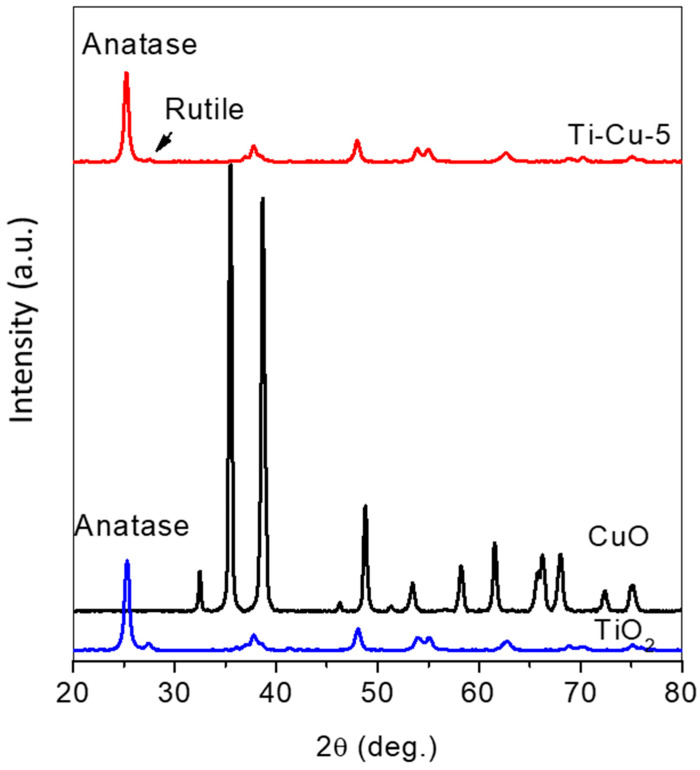
XRD patterns of Ti-Cu-5 and the corresponding single metal oxides.

Figure 1 displays the powder XRD patterns of Ti-Cu-5 along with those of the single metal oxides. The Ti-Cu-5 showed the same pattern as that of the pure TiO_2_ anatase with no signs of segregated copper oxide, indicating the well-dispersed nature of the Cu ions and no noticeable effect on the TiO_2_ phases.

Doping titanium with Cu(II) ions was found to significantly enhance the surface areas of the calcined powders. This increase can be referred to as the gel formation, which was promoted by the presence of the Cu(II) ions. The BET surface of Ti-Cu-5 was 46.3 m^2^/g compared with 29.1 for pure TiO_2_.

### 2.2. Effect of Time, pH, and Catalyst Concentration

**Figure 2:**
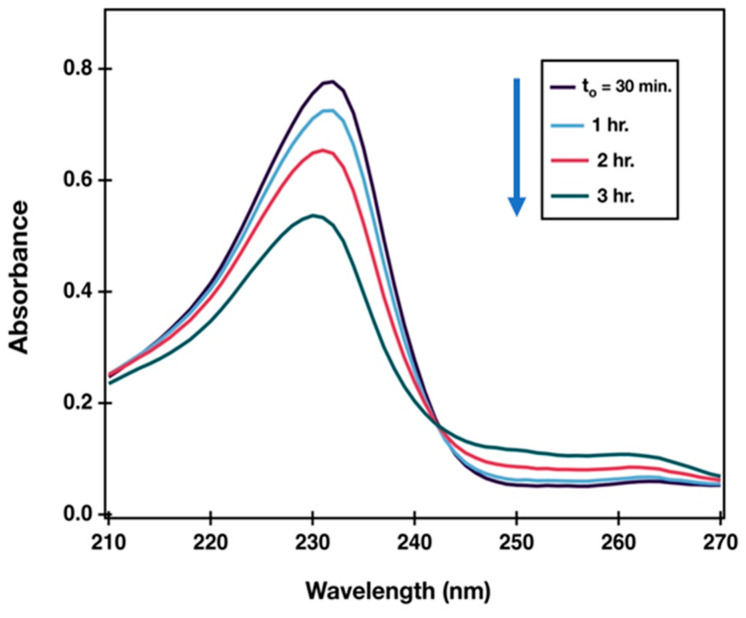
Absorbance spectrum demonstrating naproxen’s photodegradation with the optimized conditions.

Throughout the timestamps studied at the 4 h mark., the absorbance went down slightly but not considerably enough for it to be an ideal time frame for the experiment. Therefore, 3 h was taken as the optimal duration for the photocatalytic degradation. Moreover, the chosen time values may be based on prior experiments indicating the typical duration for the complete conversion or optimal yield [13]. The optimal pH condition for degradation of the 10^−5^ M naproxen solution was at an acidic pH of 4.55, where the absorbance went down considerably, and this value was thereby utilized in the last optimization experiment. The other pHs tested caused the absorbance to increase rather than decrease, indicating the lack of degradation of naproxen in those solutions. Even though the 75 mg/100 mL concentration of the 5% Cu. TiO_2_ drug yielded a greater degradation of naproxen, it was not a significant increase compared to the 50 mg/100 mL drug, so the latter was selected. Earlier reports have used comparable amount of catalysts to degrade naproxen [13]. Therefore, the optimized conditions utilized for preparing the LC-MS samples were 10^−5^ M naproxen, 50 mg/100 mL concentration of the 5% Cu. TiO_2_ catalyst, a pH of 4.55, and a degradation timeframe of 3 h in visible light, with the absorption spectra compiled and modeled by Figure 2.

Mendez-Arriaga et al. reported comparable degradation percentages of naproxen using TiO_2_ under simulated solar irradiation within 240 min [34]. Moreover, they investigated the effect of temperature, dissolved oxygen concentration, and catalyst load. They found that the maximum degradation for naproxen was observed at a TiO_2_ loading of 0.1 g/L, and increasing the temperature from 20 °C to 40 °C had a significant effect on the naproxen degradation. Moreover, the dissolved oxygen concentration was an important parameter to increase the degradation of the drug. However, it was observed that its rate of mineralization did not increase [34]. In another study, Eslami and coworkers reported the use of a nitrogen and sulfur-doped TiO_2_ catalyst, which was coated on polycarbonate (NS-TiO_2_@PC) by a simple deposition method. The photocatalytic activity of the NS-TiO_2_@PC was evaluated through the degradation of naproxen under visible-light irradiation. Their optimization results showed that maximal degradation efficiencies of 100% were achieved for naproxen [35]. However, the irradiation source was a 350 W Xenon lamp (XBO) located at the top of the reactor with variable distances. The Xenon lamp had a wavelength in the range of 380 to 900 nm.

### 2.3. Kinetics of Naproxen’s Photocatalytic Degradation

At the proposed time intervals, the variations in the absorption spectra were studied throughout naproxen’s degradation process. This yielded a photodegradation efficiency (η) of 44.77% according to the following equation [36]:

(1) $$\eta =(1-\frac{A_{0}}{A})\times 100$$

where A_0_ is the initial absorbance at 30 min, with the time spent until that particular timepoint not considered due to this representing degradation by adsorption and not photodegradation. A_t_ is the absorbance at time t, which was 3 h in this case.

(2) $$\ln(C_{t})=(-k_{app}\times t)+\ln(C_{0})\times 100$$

C_t_ represents the concentration at a certain time (t), k_app_ is the apparent rate constant, and C_0_ is the initial concentration.

**Figure 3:**
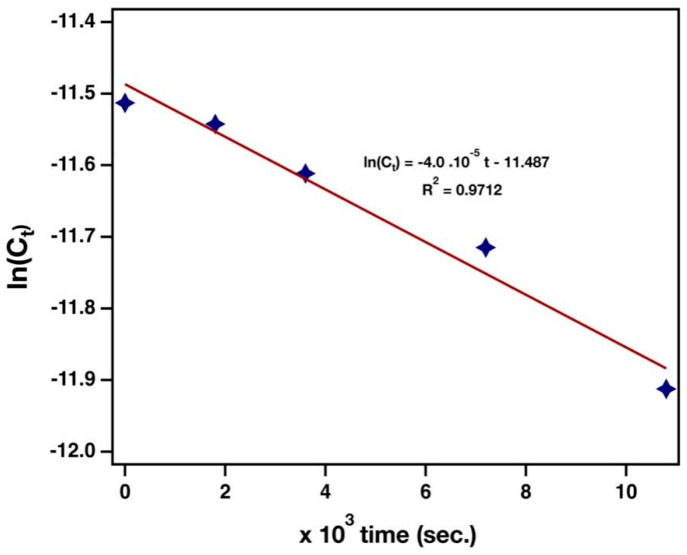
ln(C_t_) vs. time in sec., exhibiting the pseudo-first order naproxen degradation reaction.

Varying naproxen concentrations with time were studied, where the degradation process best fits the pseudo-first order reaction kinetics equation (ln(C_t_) = −4.0 × 10^−5^ t − 11.487) with a determination coefficient of 0.9712, as modeled by Equation (2) and as illustrated by Figure 3 [37].

### 2.4. Degradation Products Identification and Validation by LC-MS/MS

**Figure 4:**
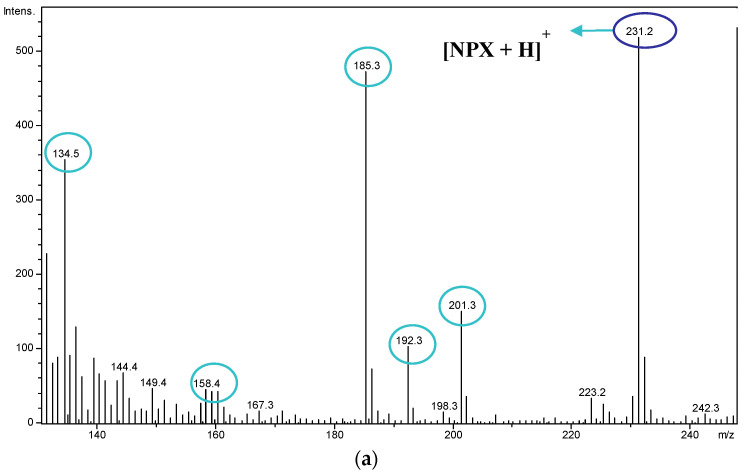

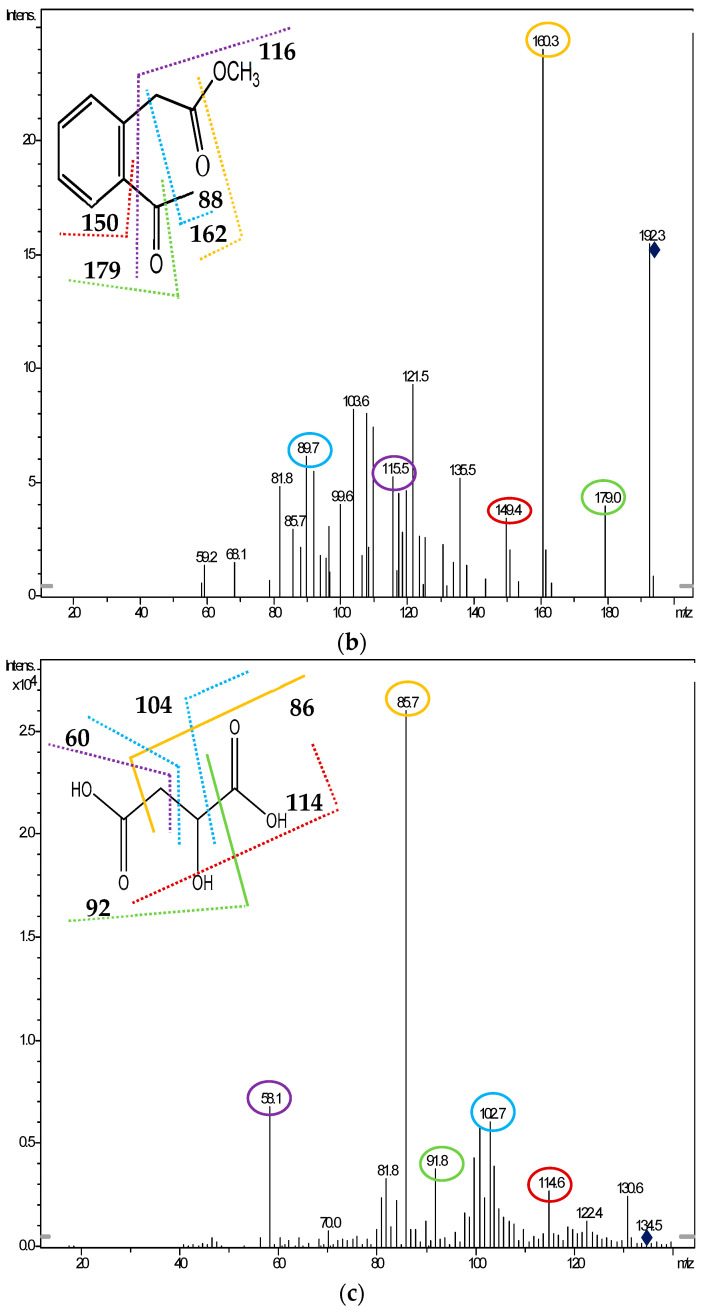
(**a**) MS spectrum of naproxen and its detected degradation products at the 1 h timestamp. (**b**) MS/MS spectrum confirming the 192 fragment’s structure. (**c**) Tandem mass spectrum of another naproxen degradation product (*m*/*z* = 134).

Multiple degradation products were detected at both the 1 h (Figure 4) and 2 h timestamps, including molecular masses at 108, 134, 158, 186, 188, 192, 201, 206, 224, 232, and 236, as well as at 104, 108, 186, 188, 202, 206, 224, 232, and 236), respectively. Both MS spectra at these time intervals exhibited peaks at 231 that refer to the protonated naproxen cation and at 253, denoting the naproxen sodium adduct—[NPX + Na]^+^—though their intensities decreased over time while the fragment intensities increased. This is further substantiated by the declining absorption spectra of naproxen shown previously in Figure 2.

These molecular weights underwent further fragmentation and produced tandem MS spectra. For instance, after 1 h of degradation, 134, 158, 186, and 192 *m*/*z* precursor ions were selected, and their structures were validated by means of the MS/MS spectra generated. Figure 4 displays the tandem MS spectra for 134 and 192. For the 2 h timepoint, 104, 122, 186, and 202 masses were isolated and fragmented as well.

### 2.5. Degradation Mechanism

Some of the identified degradation products have been reported earlier in the literature [13,24,27]. For instance, Gongduan et al. [24] reported many degradation products of naproxen when they used Bi-modified titanate nanobulk and visible-light driven photocatalytic degradation of the drug. Out of the many reported degradation products [13,24], there were three intermediates that were identical to what we are reporting in our study, including *m*/*z* 202, *m*/*z* 200, and *m*/*z* 134, which belong to 1-(6-methoxy naphthalen-2-yl) ethanol, C_13_H_14_O_2_, 1-(6-methoxy naphthalen-2-yl)ethanone, C_13_H_12_O_2_, and malic acid, C_4_H_6_O_5_, respectively. Morover, Jimenez-Salcedo and coworkers have reported similar degradation products at *m*/*z* 186 and 202 for 2-methoxy-6-vinylnaphthalene, C_13_H_12_O, and 1-(6-methoxynaphthalen-2-yl)ethan-1-one, C_13_H_12_O_2_ [27].

**Scheme 1:**
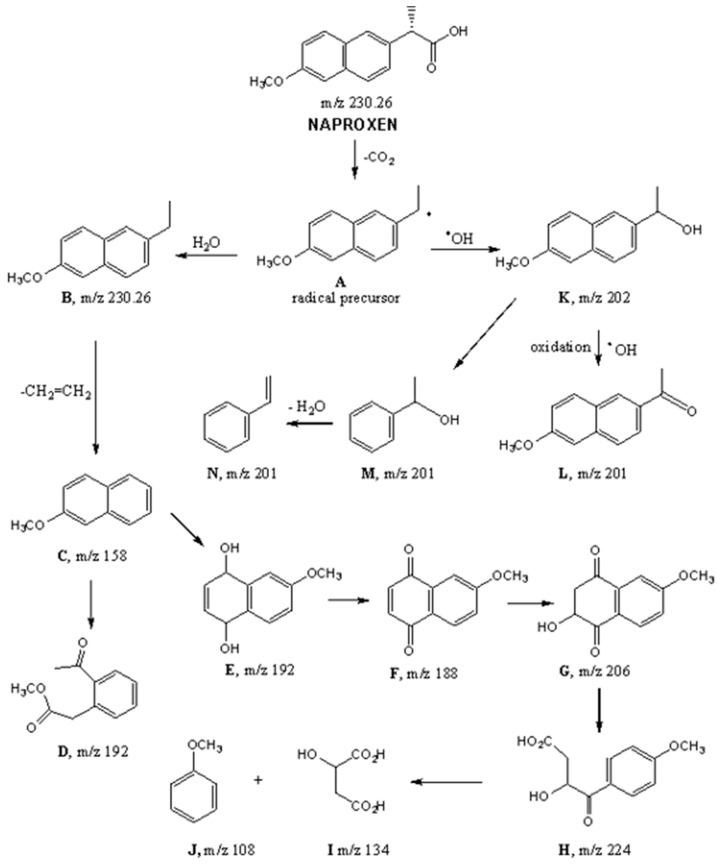
Degradation pathway of naproxen mediated by hydroxyl radicals.

**Scheme 2:**
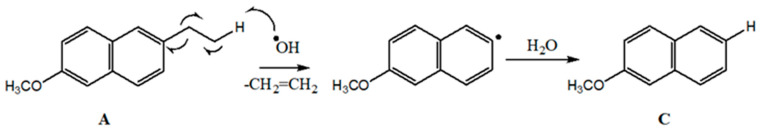
Loss of ethylene by intermediate **A**.

**Scheme 3:**
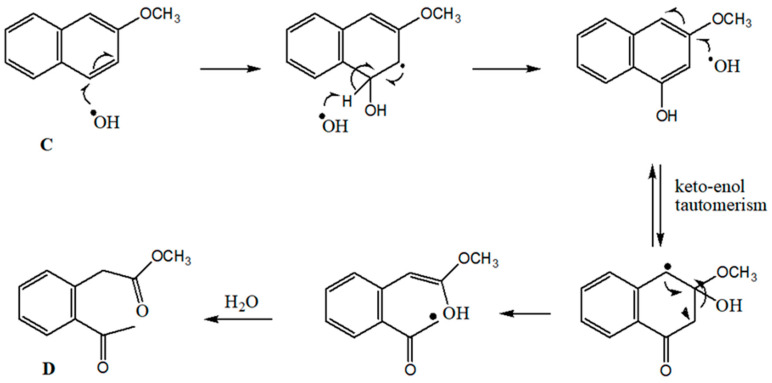
Ring opening of **C** to provide intermediate **D**.

**Scheme 4:**
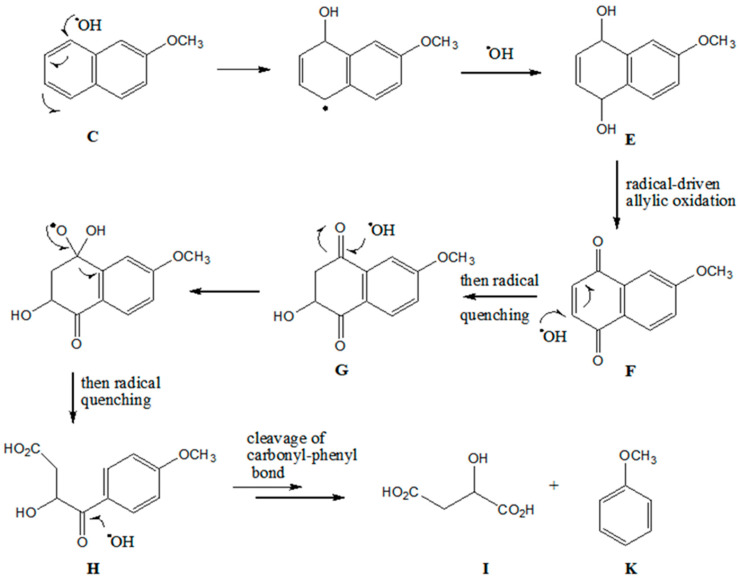
Formation of fragment **I**/**J** from intermediate **C**.

Reactive hydroxyl (·OH) radicals, produced by the catalyst in the presence of light and oxygen molecules, caused the degradation of naproxen as evidenced by the UV-Vis studies and LC-MS/MS analysis. Various intermediates were produced, and their structures were confirmed by MS/MS. Based on these structures, a degradation pathway is proposed (Scheme 1). The initial event seems to be the hydroxyl radical decarboxylation of naproxen to give a stable benzylic radical **A** from which fifteen intermediates were produced. The quenching of **A** with water and hydroxyl radicals led to the intermediates **B** and **K**, respectively. Intermediate **B** underwent the loss of ethylene, through a radical process, to produce the degradation product **C**. The mechanism for the conversion of **B** → **C** is proposed (Scheme 2). The methoxy-bearing compound **C** then undergoes a ring opening to produce the structure **D**. A plausible mechanism for the **C → D** transformation involving a series of hydroxyl radical attacks and keto-enol tautomerism is given in Scheme 3. Intermediate **C** also undergoes oxidation to the dehydroxylated compound **E**, which is further oxidized to the benzoquinone **F**, which subsequently goes through further hydroxylation to **G**, then the ring opens to **H**, and finally cleavage generates anisole **J** and hydroxy succinic acid **I**. The series of transformations from **C → I + J** are depicted in Scheme 4.

**Scheme 5:**
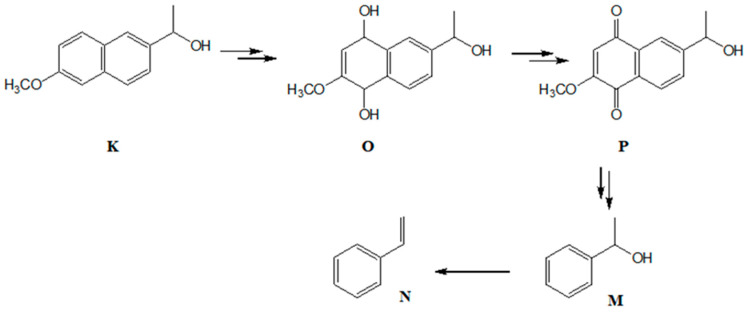
Formation of fragment **N** from intermediate **K**.

Under the reaction conditions, the alcohol compound **K** goes through a hydroxyl radical oxidation to provide the ketone **L** (Scheme 5). **K** is further degraded to the benzyl alcohol **M**, through the intermediates **O** and **P**, following a mechanism similar to that illustrated in Scheme 4, where the ring bearing the methoxy group is destroyed via oxidation to a benzoquinone intermediate. The elimination of water from the benzyl alcohol **M** then produces the styrene **N**.

## 3. Materials and Methods

The drug under investigation, namely Naproxen, has a labeled purity of more than 99%. It was obtained from Sigma-Aldrich (Saint Louis, MO, USA) and used as such. Ultra-pure water from the Milli-Q Plus system (Millipore Sigma, MA, USA) was used to make the drug solution of desired concentration.

Methanol (2-propanol), produced by Honeywell (Seelze, Germany), was obtained from Fisher Chemical, (West Chester, PA, USA), and Titanium (IV) *n*-butoxide and cupper (II) nitrate hydrate were obtained from Sigma-Aldrich (Saint Louis, MO, USA).

### 3.1. Catalyst and Gel Formulation

Six milliliters (17.6 mmol) of Ti(IV) *n*-butoxide was dissolved in 80 mL of 2-propanol. Following this, 0.92 mmol of Cu(II) nitrate was dissolved in the same amount of solvent. The Cu precursor solution was added to the Ti precursor solution, 20 mL propylene oxide (a gelation promotor) was added, and the mixture was stirred for 15 min before drop-wise addition of 1.3 mL H_2_O while stirring. The mixture was stirred for 24 h, where a bluish transparent gel was obtained. The solvent was removed from the gel by evaporation in a water bath at 85 °C. The obtained powder was dried in an oven at 120 °C for 2 h before calcination at 500 °C for 4 h.

The presence of Cu(II) had a significant effect on the gel formation process. In the preparation of pure TiO_2_, no real gel formed but rather a colloidal suspension and a very fine precipitate were formed. However, when Cu(II) ions were present in the starting solution of the Ti precursor, a transparent gel formed upon aging of the reaction mixture for around 3 days.

### 3.2. Optimization of Operational Conditions for Naproxen Photodegradation

A 10^−3^ M naproxen stock solution was prepared in LC-MS-grade methanol. A working solution, 10^−5^ M, was made by diluting the stock in ultra-pure water. This solution was then placed in a visible-light chamber containing Hg cool-white lamps s988 8W (SYLVANIA, Mexico) for 30 min with and without the presence of 5% Cu-TiO_2_. A control experiment was also performed with the copper-doped (5%) titanium dioxide in dark conditions.

After those preliminary tests were completed, varying conditions for time, amount of catalyst, and pH were examined. The catalyst concentration (50 mg/100 mL drug) and pH (4.55) were held constant, and the photodegradation of 10^−5^ M naproxen was studied at various times (0 min, 30 min, 60 min, 120 min, 180 min, and 240 min). The ideal pH was then assessed by conducting experiments at the optimal times and concentrations of naproxen and catalyst ([naproxen] = 10^−5^ M, 5% Cu. TiO_2_: 50 mg/100 mL drug) constant at neutral pH (7.55) and basic pH (9.15). The pH was adjusted by adding small aliquots of 0.1 M NaOH and monitoring the pH with a pH meter (WTW PH 3210). The concentration of the catalyst was later varied to 25 mg/100 mL of drug and 75 mg/100 mL of drug (25 below and above the typical amount). In all of these tests, the degradation process was monitored using absorbance measurements done on the SPECORD 210 PLUS spectrophotometer with solutions being filtered using hydrophilic 0.45 µm syringe Millipore filters.

### 3.3. Product Analysis and Identification Using LC–UV/Vis–MS Studies

The chromatographic experiments were carried out on a HP 1100 liquid chromatograph from from Agilent Technologies, (Palo Alto, CA, USA) using a binary solvent gradient pump and an automatic injector. The drug degradation products were separated using Agilent Zorbax^®^ SB-C18 column 150 mm × 4.6 mm packed with 5 μm particle size. The detection system was a diode array detector from from Agilent Technologies, (Palo Alto, CA, USA) with wavelength range between 200 and 780 nm. The signal acquired from the detector was recorded by HP Chemstation^®^ software (version A.06.01 on windows XP). The mobile phase consisted of two solutions, namely A and B. Solution A was made from 0.1 M of ammonium acetate and acetic acid (pH 5.2), whereas solution B was acetonitrile. The gradient elution was from 5% to 90% in 30 min, the flow rate was 0.7 mL/min, and the injection volume was 100 μL.

The gradient HPLC separation was coupled with ion trap mass spectrometer from Agilent Technologies, (Palo Alto, CA, USA). The mass spectrometer was equipped with an electrospray ionization source and operated in positive polarity. The ESI conditions were as follows: capillary voltage was 3.5 kV; endplate offset was fixed at 500 V; skimmer voltage was 40 V; trap drive voltage was set at 53 V; the nebulizer pressure was 70 psi; drying gas flow was 12 L min^−1^; and the drying temperature was 350 °C. The mass range was from 50 to 500 *m*/*z*. Tandem MS experiment was done using the Auto MS^n^ mode, wherein Helium gas was used as a collision gas.

## 4. Conclusions

The photocatalytic degradation of naproxen using 5% Cu-doped titanium dioxide was investigated and the optimal conditions were found to be 3 h, a catalyst loading of 50 mg/100 mL of the drug, and an acidic pH of 4.55. The inorganic catalyst (5% Cu. TiO_2_) was prepared by mixing the copper and titanium precursors with propylene oxide as a gelation agent followed by multiple drying cycles and, eventually, calcination. The XRD studies of the catalyst showed similarities to pure anatase, indicating an even distribution of copper inside the titanium dioxide lattice. The photodegradation of naproxen was performed under visible-light conditions with an efficiency of 44.8% and followed pseudo-first order kinetics. The degradation products of the drug were identified by LC-MS/MS for the degraded samples at 1 h and 2 h from the MS/MS fragments. The degradation pathway and mechanism were proposed based on a series of hydroxyl radical attacks and bond cleavages.

## Data Availability

All sets of data created and analyzed during this study are available via contact with the corresponding author upon reasonable request.
